# Sounding the alarm: Defining thresholds to trigger a public health response to monkeypox

**DOI:** 10.1371/journal.pntd.0007034

**Published:** 2018-12-20

**Authors:** Sarah Anne J. Guagliardo, Mary G. Reynolds, Joelle Kabamba, Beata Nguete, Robert Shongo Lushima, Okito E. Wemakoy, Andrea M. McCollum

**Affiliations:** 1 Epidemic Intelligence Service, Centers for Disease Control and Prevention, Atlanta, Georgia, United States of America; 2 Poxvirus and Rabies Branch, Division of High-Consequence Pathogens and Pathology, Centers for Disease Control and Prevention, Atlanta, Georgia, United States of America; 3 Centers for Disease Control and Prevention, Kinshasa, Democratic Republic of the Congo; 4 Kinshasa School of Public Health, Kinshasa, Democratic Republic of the Congo; 5 Ministry of Health, Kinshasa, Democratic Republic of the Congo; University of California, Los Angeles, UNITED STATES

## Abstract

Endemic to the Democratic Republic of the Congo (DRC), monkeypox is a zoonotic disease that causes smallpox-like illness in humans. Observed fluctuations in reported cases over time raises questions about when it is appropriate to mount a public health response, and what specific actions should be taken. We evaluated three different thresholds to differentiate between baseline and heightened disease incidence, and propose a novel, tiered algorithm for public health action. Monkeypox surveillance data from Tshuapa Province, 2011–2013, were used to calculate three different statistical thresholds: Cullen, c-sum, and a World Health Organization (WHO) method based on monthly incidence. When the observed cases exceeded the threshold for a given month, that month was considered to be ‘aberrant’. For each approach, the number of aberrant months detected was summed by year—each method produced vastly different results. The Cullen approach generated a number of aberrant signals over the period of consideration (9/36 months). The c-sum method was the most sensitive (30/36 months), followed by the WHO method (12/24 months). We conclude that triggering public health action based on signals detected by a single method may be inefficient and overly simplistic for monkeypox. We propose instead a response algorithm that integrates an objective threshold (WHO method) with contextual information about epidemiological and spatiotemporal links between suspected cases to determine whether a response should be operating under i) routine surveillance ii) alert status, or iii) outbreak status. This framework could be modified and adopted by national and zone level health workers in monkeypox-endemic countries. Lastly, we discuss considerations for selecting thresholds for monkeypox outbreaks across gradients of endemicity and public health resources.

## Author summary

Most of the scientific literature about statistical thresholds for outbreak detection is biased toward large datasets that derive from wealthy countries. In the scientific community, it is acknowledged that even objectively determined statistical thresholds can produce varied results when applied to the same dataset, but to date, no study has evaluated thresholds for rare but endemic diseases such as monkeypox. We applied three statistical thresholds to a monkeypox surveillance dataset from the Democratic Republic of the Congo (DRC) to identify periods of elevated disease incidence. Each threshold produced markedly different results in terms of the number of aberrant months detected. We conclude that thresholds alone are insufficient to determine when a public health response should be triggered to monkeypox, and instead propose a novel algorithm that combines the use of an objective threshold with contextual information about social and epidemiological links between cases. Similar approaches could be valuable when applied to other, rare tropical infectious diseases where data are limited.

## Introduction

Nearly forty years after the successful eradication of smallpox, monkeypox virus continues to lurk in the tropical forests of Central and West Africa. Causing a smallpox-like illness, human monkeypox was first described in 1970 in the Democratic Republic of the Congo (DRC), and is widely regarded as the world’s most important extant orthopoxvirus. Following initial transmission from an animal source, human-to-human transmission is known to occur [1], and outbreaks outside of its natural endemic range (South Sudan in 2006 [2], United States in 2003 [3]) have raised concerns about its epidemic potential. Although monkeypox is rare, many African ministries of health regard it as a disease of public health importance due to both its clinical severity and potential for transboundary spread.

In the DRC, human monkeypox is a mandatory reportable disease and has been designated by the government as one of 17 priority diseases. In the national surveillance system, all suspected cases are formally reported from 26 provinces (consisting of 519 health zones) to regional and national authorities. Current policy dictates that for each suspected case, a comprehensive response should be set in motion including a case investigation, specimen collection and laboratory testing, patient isolation, contact tracing, and community education. In practice, however, this rarely occurs. Healthcare workers are not adequately trained in case recognition, and in the face of limited resources and competing public health priorities, cases often go uninvestigated.

In response to these challenges, the DRC Ministry of Health and the Kinshasa School of Public Health in collaboration with the US Centers for Disease Control and Prevention (CDC) established an Enhanced Monkeypox Surveillance System in Tshuapa Province in 2010, which provided dedicated resources for case detection and confirmation. This resulted in a steady flow of cases that were investigated, as well as increased accuracy of case detection. A next step in the development of the system is to objectively and meaningfully distinguish between background levels of disease and abnormally high case counts (i.e., outbreaks). Currently ‘outbreaks’ are declared only when public health officials observe what they believe to be meaningful increases in case counts.

One of the tenants of epidemiologic surveillance is the timely detection of cases, yet resource limitations in endemic areas of the DRC hinder efforts aimed at rapid case confirmation and public health response. For example, complications associated with specimen transport (e.g., fuel shortages, limited flights) to the national laboratory in Kinshasa cause notable delays in diagnostic testing. Personnel turnover results in an inability to retain local healthcare workers trained in the recognition of clinical symptoms. These setbacks often translate to delayed interventions or even missed opportunities for disease prevention. With this in mind, are there objective criteria that could be leveraged to rapidly indicate a significant aberrance in case counts? And are there lessons from monkeypox that we can broadly apply to other rare diseases?

Establishing meaningful statistical thresholds for defining ‘outbreaks’ of disease may offer a solution, by objectively signaling to authorities when it is appropriate to respond to an increase in the number of observed cases. Here, we evaluate three statistical thresholds for defining outbreaks of monkeypox, propose an innovative algorithm for a public health response, and discuss the implications of using such techniques for monkeypox and other diseases.

## Methods

### Study area

Tshuapa Province is located in the Congo Basin of the DRC. It is a region with severe poverty where less than 50 physicians service a population of ~1.6 million people. In the absence of large-scale agricultural production, the population relies heavily on bushmeat as a steady source of protein [4]. There is no running water, and electricity is available only by generators. Fuel is expensive and shipped by barge to Tshuapa Province, and there are often shortages attributable to delays in shipment. This forested region has little in the way of transportation infrastructure. Public health activities are also subject to these barriers, impeding travel to rural communities in order to conduct investigations.

### Surveillance system

In the broader national surveillance system, weekly notifications of suspect monkeypox cases are delivered from local health areas to the health zone, then to the provincial and national levels. These data consist of monkeypox case counts and deaths by health zone aggregated by sex and age group. Suspect cases of monkeypox are typically identified when sick patients present to health centers. Alternatively, health care workers also may identify suspect cases in the field when conducting other health-related activities such as vaccination campaigns. Although the data reported through the national system are not detailed, notifications can lead to more rigorous epidemiological investigations and may help to generate signals of aberrance.

In the enhanced system in Tshuapa Province, healthcare workers investigate suspect cases which meet the following case definition: “vesicular or pustular eruption with deep-seated, firm pustules and at least one of the following symptoms: fever preceding the eruption, lymphadenopathy [inguinal, axillary, or cervical], and/or pustules or crusts on the palms of the hands or soles of the feet” [5]). Once a case is identified, a monkeypox-specific case report form is completed and at least two lesion specimens are collected. Specimens are sent to the Institute National de Recherche Biomédicale (INRB) in Kinshasa for testing, and the additional original specimens and an aliquot of DNA is sent to CDC for independent confirmatory testing. Case investigation and laboratory data are digitized, merged, and subsequently cleaned for analysis. Since 2010, three formal workshops for healthcare workers have been conducted, regular supervision visits have occurred, and community outreach activities have been carried out to educate locals on preventive measures and encourage patients to visit to healthcare centers.

### Comparison of response thresholds

Investigated suspect case counts from Tshuapa Province from 2010–13 (the most recent period for which cleaned, validated data are available) were used to calculate presumptive thresholds based on monthly incidence for the years 2011–2013. Although data for confirmed cases are available, we focused on suspect case counts because such data only would be available to public health officials in the context of timely warning system for monkeypox.

We followed Hay et al (2002) to evaluate three methods; Cullen, c-sum, and a World Health Organization (WHO) method, all initially developed for epidemic detection of malaria [6]. The Cullen method is typically calculated by averaging the number of cases per month for the past three years and adding 1.96 times the standard deviation, thereby constructing the 95% upper confidence limit for cases [7]. Our limited data meant having a much lower probability of identifying extreme observations. Therefore, we modified this approach to identify the upper 70% of observations (mean + 1.036*SD). The cumulative sum (c-sum) method relies on the construction of a “base year” by averaging case counts for the month of interest with estimates from the previous and subsequent months [8–10]. The threshold itself is then calculated by computing a ratio of current to past cases, where ratios exceeding one represent unusual increases in disease incidence. Lastly, the WHO method uses the upper 3^rd^ quartile of data from past cases to identify elevated instances of case counts based on retrospective monthly case data [11]. With this approach, quartiles are defined as follows: quartile 0 is smallest observed case count in the dataset, quartile 1 is the second lowest, quartile 3 is the second highest, and quartile 4 is highest observation in the dataset. The threshold is crossed when the number of observed cases for the current month exceeds the second highest observation for that month based on retrospective data. For each year the thresholds were calculated, we included all available past data in addition to data for the current year. For example, to generate thresholds for the year 2013, we relied on incidence estimates for 2010, 2011, 2012, and 2013. A WHO threshold was not calculated for the year 2011 because of insufficient data.

The Cullen and c-sum methods rely on calculation of the arithmetic mean, which is a valid measure of central tendency for normally distributed data. We tested for non-normality with Shapiro-Wilk tests to evaluate whether raw case counts or log-transformed data should be used for these approaches.

When the observed cases exceeded the threshold for a given month, that month was considered to be an ‘aberrant’ month. Thresholds were then compared by summing the number of aberrant months for each year and method. We also made month-by-month comparisons to determine whether each method identified the same months as being irregular. Finally, we consider key qualitative characteristics of each threshold (ease of calculation, resources required for calculation).

## Results

### Baseline data

Observed case counts ranged from a minimum of 6 cases (May and June 2011) to a maximum of 59 cases (May 2013), with a mean of 26.4 cases per month across all three years (SD = 15.6). The overall number of observed suspected cases was highest in 2013 (mean = 34.4, SD = 17.3) in comparison with the previous two years (2011 mean = 18, SD = 13.9; 2012 mean = 26.8, SD = 11.6). We found little evidence of skewness in the data, and, therefore relied on the raw data for the calculation of the c-sum and Cullen methods (Shapiro-Wilk tests p > 0.1, with the exception of cases counts for the month of August, W = 0.75, P < 0.0001).

**Fig 1:**
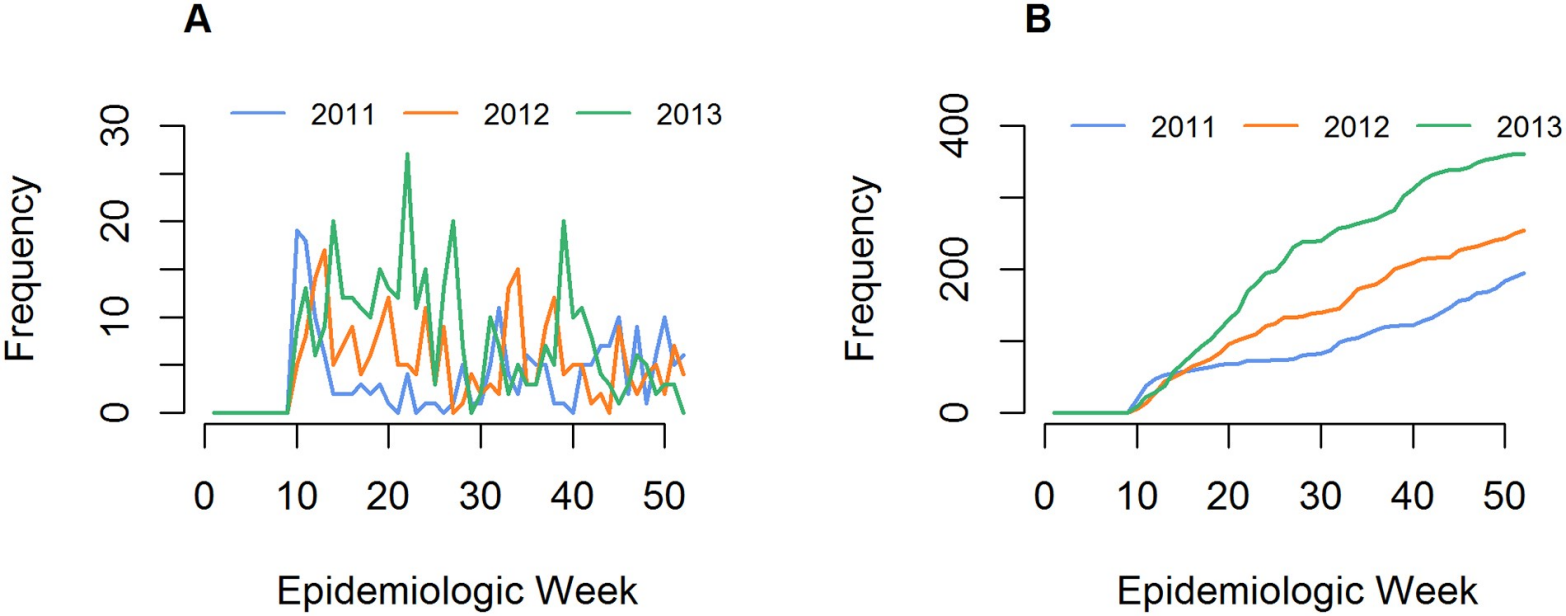
Suspected monkeypox cases in Tshuapa Province, 2011–2013. Case counts by epidemic week (A) and cumulative case counts (B) for the study years. Under the current operational scheme, health care workers anecdotally note when there is an unexpected increase in cases without systematically considering past case counts.

In 2011, the highest case counts were observed in March, followed by the period August through December (Fig 1). Data from 2012 showed a distinct pattern: although there were also high case counts in March, suspected cases for January-February and April-June were notably higher. A second peak in case counts was observed in August-September. In 2013, the period April through July demonstrated increased observed suspected cases, followed by a decline until an upsurge in October. When averaging across all three years of data, monthly case counts were highest during the months of March through June (3-month average = 108.7), and lowest in July (53), followed by November-February (4-month average = 61.8).

### Comparison of findings by method

The Cullen method produced the highest threshold values, and accordingly detected the least number of aberrant signals over the period of consideration (9/36 months or 25%). In contrast, the c-sum method resulted in the lowest cut-off values overall, and was the most sensitive approach, resulting in 30/36 aberrant months (83%). The WHO method resulted in 12/24 aberrant months (50%).

**Fig 2:**
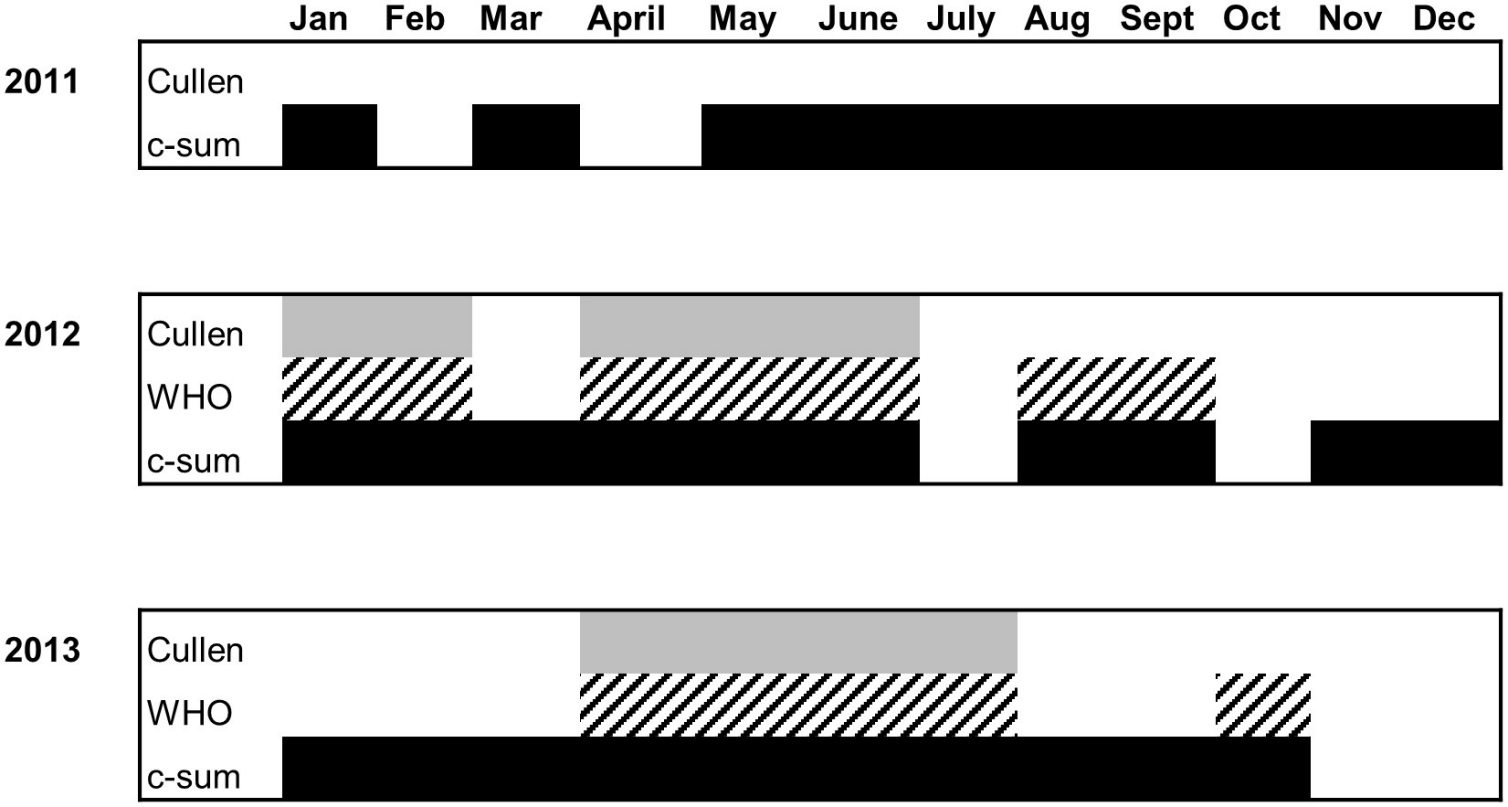
Aberrant months detected by method, 2011–2013. Aberrant months are indicated by shaded blocks for each month: Cullen (light grey), WHO (hatching), and c-sum (black). The WHO method was not applied to 2011 suspected monkeypox case counts due to insufficient historical data.

In 2011, only c-sum resulted in detection of aberrant months (all months except February and April); the Cullen approach did not detect any aberrant months. In 2012, congruence across all methods was observed for the months January, February, April, May, and June (50% agreement, out of 10 total aberrant months across all methods). Agreement was observed in 40% of months (4/10) in 2013 (April, May, June, and July) (Fig 2). Although some months were consistently deemed abnormal by all three methods across all years (30%, 9/30 aberrant months), there was no discernable seasonal pattern.

### Ease of calculation by method

The WHO approach involves the most straightforward calculation that could easily be calculated by hand, advantageous when limited resources (human or computational) are available (Table 1). The Cullen and c-sum methods, in contrast, may require log-transformations on non-parametric data; this may be cumbersome in areas where computers are less accessible. The Cullen method could be performed using Microsoft Excel. C-sum involves the most complex calculation, requiring averaging of case counts from several months, in addition to the calculation of a ratio of past to present months. The latter method could also be calculated in Excel, but would require a more in-depth understanding of data manipulation.

**Table 1:** Summary of methods.

	WHO	Cullen	C-sum
**Calculation**	*x*_*n*-1_ Where, *x* = the highest observed number of cases for a given month, over the course of several years	*μ + 1*.036*σ_*X*_ Where, *μ* = the mean number of cases for a given month *σ* = the standard deviation of cases for a given month	*x*_*n*_/((*i*_*n*-1_ + *i*_*n*_ + *i*_*n*+1_)/*I*) Where, *x* = the number of cases for a given month for the current year *i* = the number of cases for a given month for past years *I* = the number of months considered (i.e., 3 months)
**Complexity of calculation**	Easy	Medium	Difficult
**Resources required for calculation**	Could be computed by hand	Log-transformation much easier with statistical software or spreadsheet	Log-transformation and moving window much easier with statistical software or spreadsheet
**Sensitivity**	Moderately sensitive	Least sensitive	Most sensitive
**Best application**	Resource-constrained settings Small datasets Rare disease	Larger datasets (>30 observations/time unit) Non-rare endemic disease	Larger datasets (>30 observations/time unit) Datasets with temporal stochasticity

Each of the three thresholds are evaluated on the basis of ease or complexity of calculation, resources required for calculation, and sensitivity.

## Discussion

### Response thresholds for monkeypox

The true reservoir of monkeypox remains a mystery, and data generated through epidemiologic surveillance are among the few tools available to address lingering questions about its transmission dynamics in humans and in nature. Still, the scientific literature about surveillance algorithms is biased toward the reporting of rare diseases in developed nations, with data most commonly aggregated at weekly timescales [6, 8, 12–16]. Because these approaches are unsuitable for remote regions where electricity is scarce and data are often stored on paper forms, in this analysis we intentionally evaluated only thresholds that are relatively straightforward to calculate and conceptually easy to understand.

The WHO method is the most intuitive of the three methods considered, but in contrast to c-sum, it is hypersensitive to variation in case counts between months. WHO therefore might be most relevant for rare diseases with low baseline case counts (small datasets) for which the mean as a measure of central tendency is untenable. The Cullen method, however, does utilize the calculation of the mean, and so is most suitable for larger datasets (>30 units/time unit). As the least sensitive approach, Cullen might be advantageous for identifying extreme instances of elevated cases counts for diseases that are typically rare, but that sometimes result in explosive outbreaks (e.g., measles). Lastly, the c-sum approach relies on the averaging of case counts from the previous and successive months, resulting in a ‘smoothing’ effect on the data. In essence, this allows for detection of aberrant events when monthly case counts fluctuate because of either biological stochasticity or systematic unreliability. As an example of the former, periodicity in monkeypox case counts might occur because of fluctuations in climatic variables, which in turn influence host populations and contact patterns between humans and nonhuman reservoirs. Alternatively, surveillance systems themselves may also experience momentary disequilibrium. In Tshuapa, tireless health care workers are at times shifted from regular activities to attend to immediate demands of (for example) childhood vaccination campaigns or trainings, and, thus, are not available for monkeypox surveillance work. Such changes in workforce are known to have affected the intra-annual variation in the present data.

Similar to previous findings [6], each threshold produced markedly different results. Which then is the most reliable or appropriate for identifying monkeypox outbreaks in Tshuapa Province? Some insight is offered by the tiered approach proposed in the Integrated Disease Surveillance Response (IDSR) guidelines for diseases like polio and measles: an alert threshold first prompts further investigation/intervention beyond routine surveillance to verify the occurrence of an abnormal situation, and a second epidemic threshold then triggers more forceful action such as contact tracing and community education [17].

**Fig 3:**
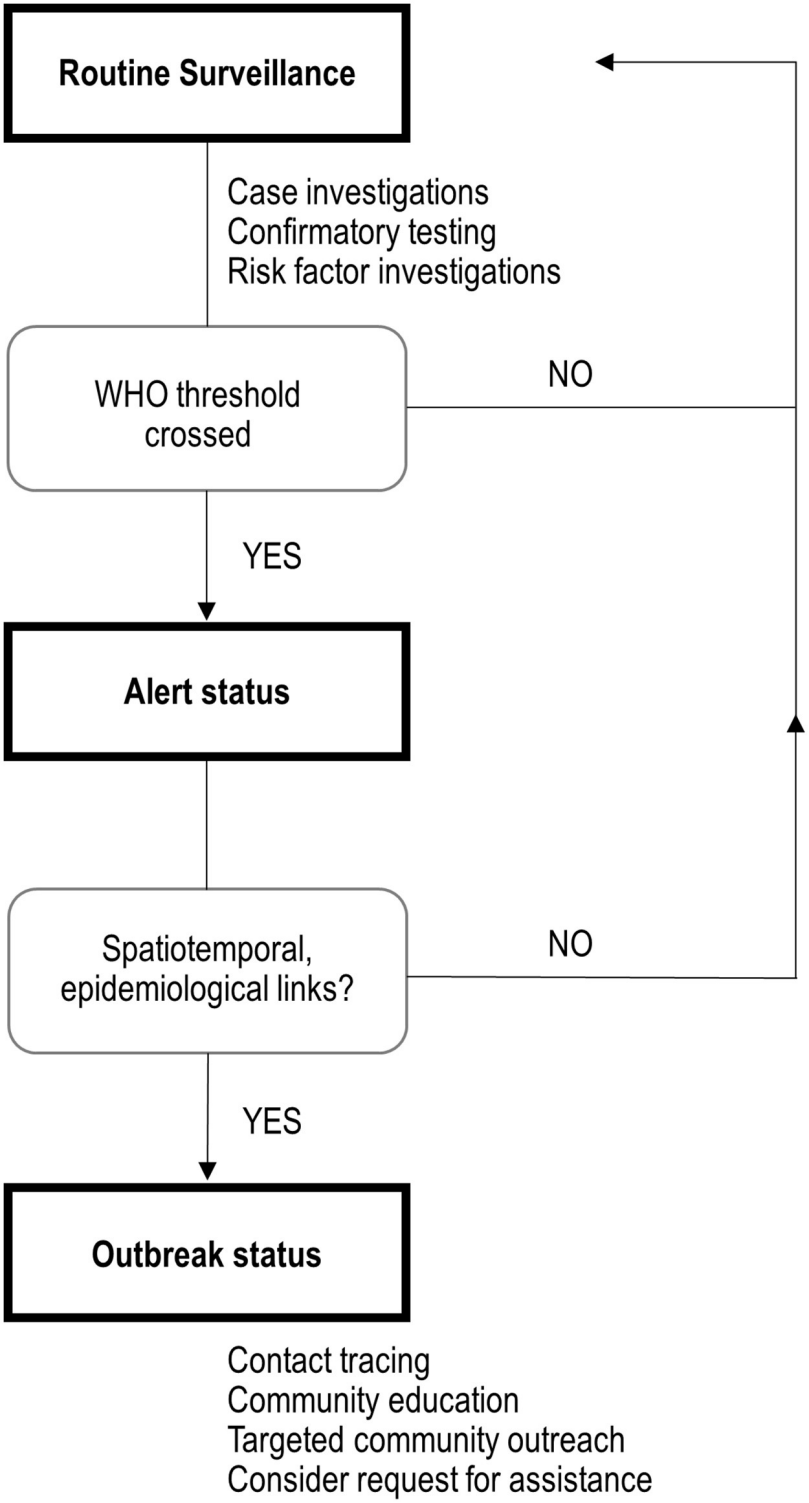
Proposed monkeypox response algorithm in Democratic Republic of Congo. We propose a novel algorithm consisting of three tiers: routine surveillance, an alert status, and an outbreak status. Elevation from routine surveillance to an alert status occurs when the WHO threshold is crossed, defined as the second highest number of cases per month for a given health zone. At this point, health care workers must investigate further to determine whether there are spatiotemporal or epidemiological links between observed cases. If yes, an outbreak status is declared, possibly entailing contact tracing, community education and request for external assistance, in addition to routine surveillance activities.

For countries where human monkeypox occurs routinely, we propose an algorithm that integrates this tiered approach with objective thresholds (Fig 3). The WHO method (upper 3^rd^ quartile of data, defined as the second highest observation) is the trigger for the first of these levels due to its ease of calculation and intuitiveness. Public health workers at the health zone level in the most remote parts of the region can rapidly calculate this threshold. Once the threshold has been crossed and an alert status declared, health zone authorities then direct local healthcare workers to investigate the temporal and the geographical delimitation of the problem, and establish whether there are epidemiological links between cases. For example, cases occurring within a similar timeframe (within one month), in a similar geographic region (within 100km), and where there is evidence of social and/or epidemiological links between cases (e.g., family members or bushmeat obtained from the same source). If at least two of these criteria are satisfied, more intensive action may be necessary including contact tracing, community education, and healthcare worker trainings. At this point, consideration could be made for assistance and mobilization of resources, stemming from either within the country (e.g., Ministry of Health) or from international sources [18].

### Considerations for MPX response thresholds in different settings

**Fig 4:**
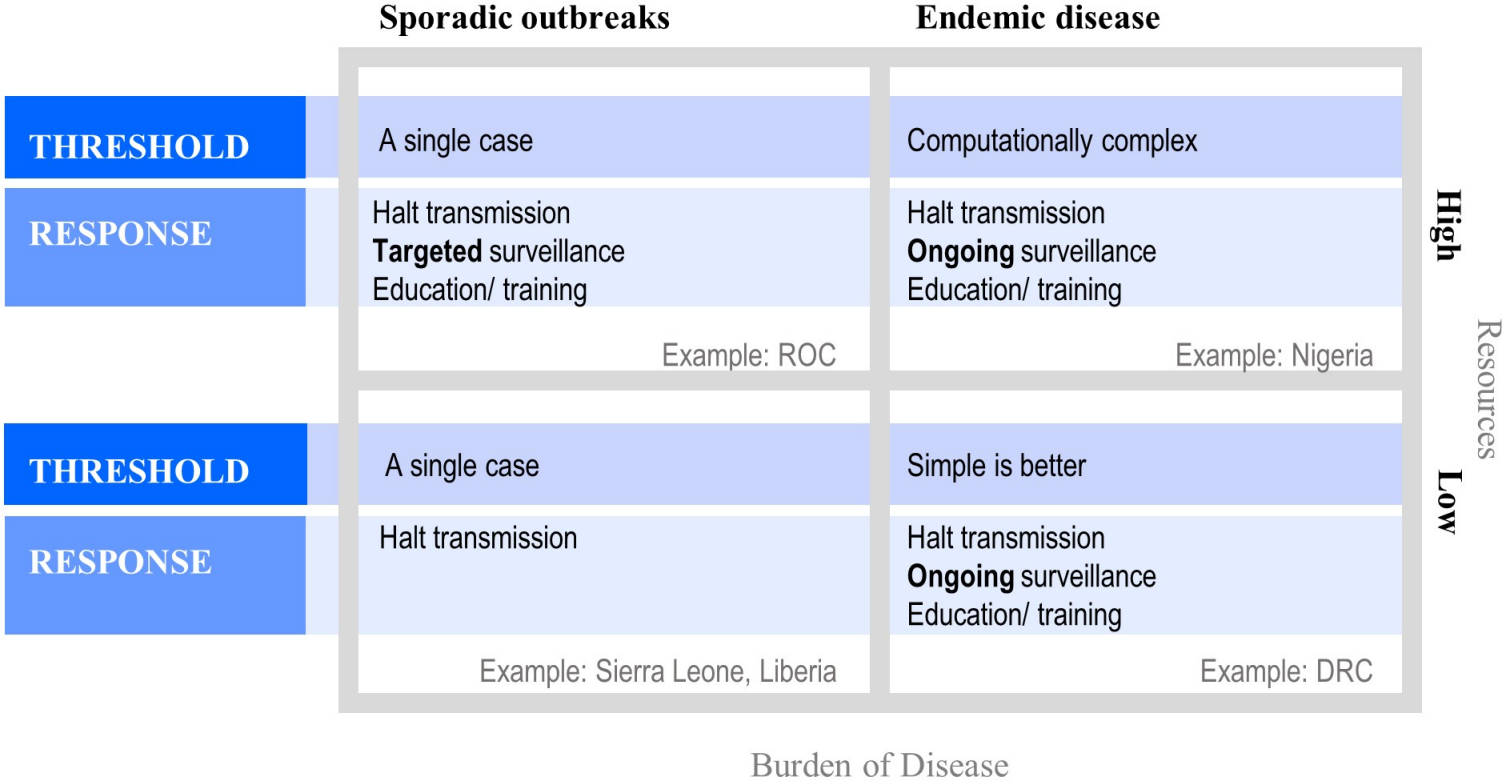
Considerations for thresholds and response goals across gradients of endemicity and resources available for public health. Thresholds for defining aberrant disease events are most relevant in the context of endemic disease; when disease is rare, contrastingly, a single case can serve as an alarm. Response goals should be prioritized—the minimal goal is always to halt transmission, but when resources are available and/or burden of disease is high, public health authorities should strive to improve surveillance and education. For monkeypox, high endemicity and low resources are exemplified by Tshuapa Province, DRC. Nigeria might be an example of high burden of disease and ample public health resources—recently confirmed monkeypox cases indicate frequent autochthonous transmission, but more data is required to determine the true burden of disease. Nigeria is currently in the process of laying the groundwork for long-term monkeypox surveillance programs—a major contrasting point with Tshuapa Province is better access to real-time case confirmation. Low monkeypox endemicity might be exemplified by the high-resource Republic of the Congo (ROC) and low-resource Liberia or Sierra Leone. In this framework, resources available for public health are defined as the general domestic health expenditures per capita.

The intensity of disease detection and response is driven by both the burden of disease and availability of public health resources, which could be measured by detected cases and per capita domestic expenditure on health, respectively [19] (Fig 4). In a high-endemicity and low-resources setting, simple thresholds are preferred. A costly surveillance system can do more harm than good by unintentionally forcing health care workers to spend salaried time collecting, cleaning, and analyzing data, drawing them away from their regular duty to treat sick patients [20]. Response thresholds in this region, therefore, should satisfy the immediate need to identify and contain outbreaks, possibly at the expense of sensitivity. Long-term investments in ongoing surveillance, healthcare worker education, and community education are also important components of prevention.

When public health resources are ample and infrastructure more developed, an abundance of well-trained data analysts offer the potential for development of complex and innovative response algorithms that more precisely detect aberrant events. Similar to any endemic setting, thresholds in this scenario should be developed with the motivation to halt disease transmission and invest in routine surveillance and education.

In low-endemicity settings, long transmission chains are rarely observed, rendering outbreak thresholds irrelevant—that is, a single case of monkeypox should be a sufficient alert signal. Instead, more consideration should be devoted to public health response activities like containing transmission, targeted education for affected community members, and healthcare worker trainings.

### Limitations

We acknowledge some limitations of the analysis. First, the thresholds were developed under the assumption of access to large retrospective datasets to reliably produce monthly case count estimates, yet the data were limited to just three complete years. It would be valuable to repeat this analysis for other DRC regions outside of Tshuapa Province, and when more data become available for recent years. Secondly, over time, healthcare workers have improved at accurately identifying cases; the predictive value positive for monkeypox cases improved markedly from 35% in 2012 to nearly 80% in 2013. Several research studies were also initiated in 2013 in three health zones, influencing the ability of healthcare workers in these health zones to launch investigations and correctly identify monkeypox cases. As a result, more aberrant months were consequently detected in 2013 likely as an artifact of surveillance quality, rather than a true increase in the observed number of cases. We are also unsure as to whether intra-annual fluctuations in case counts are real and due to biological seasonality, or whether they could be a result of more frequent reporting periods, when public health workers are present at their health zones and not away for other work-related matters.

### Conclusions

The proposed algorithm could be adopted throughout DRC, but carried out at the health zone level. A pilot period could perhaps be first be implemented in Tshuapa Province, followed by implementation in other monkeypox-endemic regions. Ideally, the response algorithm would be integrated with similar algorithms for other rare but concerning diseases, such as Ebola–trainings held at both the provincial and health zone levels could also help ensure both case recognition and the standardization of the approach across regions.

Statistical thresholds cannot broadly be applied to different epidemiological and socioeconomic contexts, as each scenario presents its own set of constraints. Consideration should be given to the resources required for calculation, sensitivity, and appropriateness of the measure in relation to underlying disease transmission dynamics (i.e., seasonality). Although we use monkeypox data to explore objective thresholds for public health response, many of the lessons drawn from this exercise could be more widely applicable to other rare, endemic diseases.
